# Numerical and Experimental Investigation of Oil Palm Shell Reinforced Rubber Composites

**DOI:** 10.3390/polym12020314

**Published:** 2020-02-03

**Authors:** Subhashini Anandan, Cuin Yang Lim, Boon Thong Tan, Vivi Anggraini, Mavinakere Eshwaraiah Raghunandan

**Affiliations:** 1 Civil Engineering Discipline, School of Engineering, Monash University Malaysia, Bandar Sunway 47500, Malaysia; subhashini.anandan@monash.edu (S.A.); cuinyang97@gmail.com (C.Y.L.); vivi.anggraini@monash.edu (V.A.); 2 Mechanical Engineering Discipline, School of Engineering, Monash University Malaysia, Bandar Sunway 47500, Malaysia; tan.boon.thong@monash.edu; 3 Advanced Engineering Platform, Monash University Malaysia, Bandar Sunway 47500, Malaysia

**Keywords:** hyperelastic model, strain energy function, compressive loading, finite element

## Abstract

This paper presents a pioneering effort to ascertain the suitability of hyperelastic modelling in simulating the stress–strain response of oil palm shell reinforced rubber (ROPS) composites. ROPS composites with different oil palm shell contents (0%, 5%, 10% and 20% by volume) were cast in the laboratory for the experimental investigation. ROPS specimens with circular, square, hexagon, and octagon shapes (loading surface) were considered to evaluate the accuracy of finite element simulation considering the shape effect of composites. Strain-controlled (compressive) tests with ε ≈ 50% at 0.8 Hz frequency were conducted in the laboratory and the test data obtained was used as input to simulate material coefficients corresponding to the strain energy functions chosen. Five different strain energy functions were selected and utilized for the hyperelastic modelling in this study using finite element approach. The shape effect was then used to ascertain any variation in the simulation outcomes and to discuss the effect of shape on the behaviour of ROPS composites in comparison to existing literature. The numerical predictions using the Yeoh model (error ≤ 2.7% for circular shaped ROPS) were found to perform best in comparison with the experimental results, thus a more stable and suitable hyperelastic model to this end. The Marlow (error ≤ 4.6% for circular shaped ROPS) and Arruda Boyce (error ≤ 4.7% for circular shaped ROPS) models were amongst the next alternatives to perform better. Even with the other shapes considered in this study, Yeoh, followed by the Marlow function, were more appropriate models. The shape effect was then studied with particular emphasis on comparing and assessing them with that observed in the literature. To this end, adopting the Yeoh function in the finite element model is the ideal approach to estimate the stress–strain response of ROPS composites.

## 1. Introduction

Rubber composites with natural fibres have seen increasing usage over the previous decades, with noted applications in the automobile industry, sports and construction sectors [1,2]. Industrial development utilizing natural fibres in rubber composites indicates a consistent growth worldwide [3]. This likeliness and preference of composites with natural fibres over synthetic fibres, are mainly oriented with their sustainable and eco-friendly nature [4], in addition to other merits including their lightweight, low cost, flexible processing and abundance in availability, making them more appealing and affordable [5]. By definition, natural fibres are typically extracted or derived from plants, animals and/or geological processes [6]. As far as their utilization in the rubber composites is concerned, coir, bamboo, sisal, jute, hemp and oil palm fibres are amongst the commonly used fibrous materials as frequently encountered in the literature [7,8,9]. On the other hand, the shock and/or vibration absorption potential of natural shells—such as coconut and oil-palm shell—owing to their high strength-to-flexibility ratio and controlled dampening nature have been recently investigated and discussed in the literature [9]. This is obviously advantageous and a pioneering option to prepare rubber composites with enhanced shock absorption behaviour in addition to their improved mechanical properties to resist compressive loads/deformations and as appropriate.

Oil palm shells (OPS) are amongst various biomass produced in the extraction process of palm-oil and their derivatives, with an abundance of availability in Malaysia [10]. OPS typically demonstrate diverse structural properties, of which the shock absorption property due to their dome-shaped structure is of utmost significance in this study [11,12]. It is perhaps fair to expect OPS to have a high energy dissipation capacity, similar to that of coconut shells [13], considering that the OPS and coconut have a similar structural texture. The good insulation properties of OPS due to their air-entrapped high porosity [14] is another added advantage for rubber composites. Nonetheless, comparison of shell with fibres to exhibit similar reinforcement or load-bearing mechanisms can be certainly debatable—particularly due to the unavailability of cable type structure in shells. Notwithstanding this, a recent study revealed sufficient enhancement in the tensile strength of an unsaturated polyester resin when reinforced with OPS [15]. However, the key point of consideration in this study is the performance of OPS reinforced rubber composites (ROPS) when subjected to repeated compressive loads or deformations (strains), thereby giving the least consideration to the properties of the composite under tensile conditions. In this view, a majority of the literature encountered focuses on investigating the suitability of other forms of biomass from oil palm tree for reinforcing rubber composites [1,8,16] and their behaviour under tensile conditions. However, very limited studies relevant to ROPS and their mechanical characterization under compressive loads, with emphasis on their stability and shock absorption natures, were encountered in the literature search. Addressing this research gap is the main focus of the work presented in this paper.

Adopting extensive field and laboratory experimentation is perhaps the most obvious method to move forward with assessing the performance of ROPS under compressive repeated loads. Nonetheless, such an approach could be expensive—considering the time, facility, and cost. Thus, numerical simulations to estimate the behaviour of ROPS is the best alternative. This, however, possesses another challenge, which is to select the most suitable model and approach for numerical estimation. Earlier, a simplistic tendency was to model rubber-like materials using only linear behaviours. This approach may not suffice because the typical behaviour of rubber is elastic and highly non-linear, which is ideally characterized as hyperelastic behaviour [17]. Even previous experiences using complex finite element (FE) analysis tools by adopting elastic and/or elastic-plastic material models are reported to show very low confidence in simulating the actual behaviour of rubber-like material [18]. Most of these limitations are addressed by adopting hyperelastic models, which can efficiently study the stress–strain response of rubber and rubber composites under real time loading scenarios. They typically involve a mathematical description of the stress–strain response of the material [19], which is often used to design the hyperelasticity of rubber rather than the Young’s modulus and Poisson’s ratio [20]. These mathematical functions are denoted as strain energy functions (SEFs). The pioneering work on hyperelastic modelling dates back to the 1940s, involving a phenomenological approach to derive the non-linear stress distribution of rubber from a continuum mechanics viewpoint [20]. However, this model was found unsuitable for compressive deformations. Since then various strain energy functions are continuously being developed in order to determine the stress strain behaviour of hyperelastic materials that can best fit the experimental data [21,22]. For example, the Hyperelastic Arruda Boyce model was used to simulate the behaviour of rubber-organomodified kaolin composites [23], and the tensile properties of Arenga Pinnata fibre-reinforced silicone rubber were predicted effectively using the hyperelastic Neo Hookean model with relatively less error [24]. In another work, with modifications to the existing hyperelastic models, a constitutive model was proposed for the tensile testing of oil palm mesocarp fibre [25]. To this end, it is clearly evident there is a sufficient lack of investigation addressing the utilization of hyperelastic models for ROPS or equivalent rubber composite types. Especially, with very limited (perhaps no) studies ascertaining the numerical simulation of ROPS or equivalent type composites subjected to compressive loads or deformations were encountered in the literature, which is the key focus of this paper.

The main aim of this study, therefore, is to estimate the behaviour of ROPS specimens subjected to compressive strains using FE simulations aided with different hyperelastic SEFs which are pre-selected based on their suitability as evaluated in comparison with the laboratory findings from trial ROPS samples. This aim was achieved using the following specific steps: Firstly, circularly-shaped (top surface) ROPS specimens were prepared in the laboratory with varying OPS contents (0%, 5%, 10% and 20%). The test specimens were then subjected to repeated compression (50% strain) tests at 0.8 Hz frequency. The stress–strain responses measured in the tests were used to calculate the elastic modulus and Poisson’s ratio values. Similar efforts and procedures were used to cast ROPS specimens with square, hexagon and octagonal shapes. Different shapes considered in this study are used to ascertain any variation in the FE simulation outcomes and to evaluate/discuss the effect of shape on the behaviour of composites with reference to existing literature. The experimental findings are then used to simulate the ROPS specimens effectively using the hyperelastic functions in the ABAQUS FE software package in order to find the best SEF that can provide an effective resemblance between the model predictions and measured stress–strain data. Since the rubber composite is hypothesized to serve well under compressive loads, the uniaxial compression type deformation mode was given key consideration in this study. The mechanical behaviour of ROPS specimen is determined in terms of their maximum compressive stress (σ_max_) under the effect of varying OPS contents. The tensile stress applications were also simulated to compare and qualitatively validate the model behaviour with previous literature. The outcomes generated from this study—stress distribution and deformation patterns—due to varying OPS content and shape effects are then discussed to ascertain the main aim of this paper in the conclusions. This study, therefore, serves as one of the pioneering studies to characterize and simulate the behaviour of ROPS or equivalent rubber composites subjected to repeated compression loading (strains).

## 2. Experimental Procedure

The ROPS specimens used in the experimental investigation of this study were cast in the laboratory. The two main materials used in casting the composites included a commercially available liquid rubber (Flex Seal, Weston, FL, USA, www.flexsealproducts.com) and OPS. OPS samples collected from local oil palm mills in Peninsular Malaysia were initially washed to remove impurities and dried to room temperature. The OPS samples were then segregated by sieving, to ensure only the samples passing sieve of 4.75 mm opening were collected. Thus-obtained rubber and OPS samples were then used to cast the ROPS specimens to the desired dimensions. The casting process involved, pouring the synthetic rubber sealant (in liquid form) into the respective moulds with continuous and gentle dispersion of the OPS in the rubber matrix. The OPS content was varied at 0%, 5%, 10% and 20% of the finished ROPS specimens measured by the volume of two components of the specimen. Thus, the weight of OPS used to attain 5%, 10% and 20% specimens were 1.75 g, 3.5 g and 7 g, respectively.

**Figure 1:**
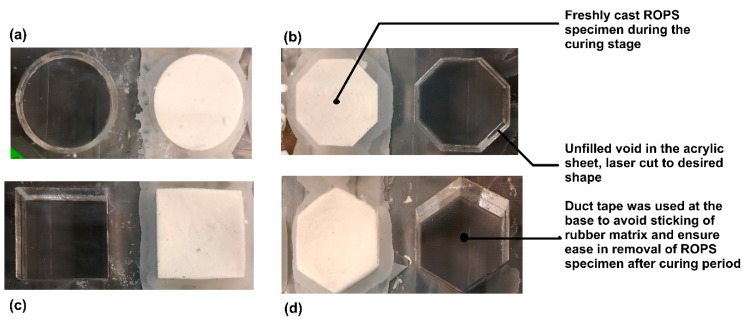
Images showing the moulds used for casting the ROPS specimens in laboratory: (**a**) circular; (**b**) square; (**c**) hexagon; (**d**) octagon.

The moulds basically determine the accuracy in the dimensions of the ROPS specimens achieved after casting. Thus, enough care was exercised in preparing the moulds. With no specific standards for testing ROPS composites yet, the diameter and thickness of circular ROPS specimens were deemed appropriate as per the D/H ratio recommendations by David Cole [26]. The dimensions of the ROPS specimens with other shapes were determined based on the equivalent area of the circular specimens. This means that the dimensions for other shapes were calculated such that the surface area remains the same as that of the circularly-shaped specimens. The circular specimens were cast to a surface diameter of 39 mm, thus, the side length for the square-, hexagon- and octagon-shaped ROPS specimens were 34.6 mm, 21.4 mm and 15.7 mm, respectively, whereas the thickness of the ROPS specimens were fixed to 20 mm throughout. Acrylic plate was laser-cut to the respective sample dimensions to cast the ROPS specimens. Figure 1 shows images captured during one of the casting processes in this study, with one mould (all shapes) filled with the rubber matrix while the other is empty for demonstration purposes. The so-cast specimens were set aside to attain undisturbed curing for a period of 14–28 days at room temperature, before being used for testing to determine their stress–strain response under repeated compressive loading. Thus, 16 ROPS specimens (four specimens on each shape) were cast in triplicates to ensure repeatability in the test results, thereby totalling to 48 ROPS specimens being used in the experimental investigations of this study. 

The main purpose of the laboratory testing is to measure the stress–strain response of the ROPS specimens when subjected to repeated compressive loading conditions. For this purpose, the universal testing machine of INSTRON make, equipped with a load cell of 50 kN maximum capacity was used. A strain-controlled test was conducted to a maximum strain of 50% at 0.8 Hz frequency (approximating to a displacement rate of 120 mm/min). The input of these test specifications were controlled through the software supplied within the equipment, which logged the specified test outputs as well. Each loading cycle basically involved compressing the specimens to strains close to 50% followed by unloading back to the original position (zero strain). The loading cycles continued up to 100 cycles and the maximum compressive stress values were recorded from the hysteresis loop of every cycle. Further, using the stress–strain data, Young’s modulus values were calculated accordingly [19]. In addition to the load cell readings, vertical and lateral displacements were recorded in order to calculate the Poisson’s ratio. Though temperature has a significant influence on the stiffness of rubber like materials [19], all the tests in this study were conducted at room temperature and, basically, the effect of temperature was not considered in this study. Amongst the key observations during initial test trials, the ROPS specimens were observed to laterally displace from the loading position with progress in the number of loading cycles, leading to erroneous load-displacement reading. This practical issue was addressed by considering an acrylic plate to hold the samples in position, to prevent them from sliding away during the loading cycles.

## 3. Numerical Studies

FE simulation in this study was conducted using the hyperelastic models in the commercially-available ABAQUS FE package (version v2018, Dassault Systèmes, France). Hyperelastic models available in ABAQUS are proved suitable to model artificial rubber components used for various applications like shock pad, rubber tire, etc. [27,28,29]. To initiate the hyperelastic modelling of ROPS specimens, it is important to first develop a representative stress–strain data and then find the appropriate SEFs. In ABAQUS, the least square fit method was adopted to curve fit the SEFs with the measured stress–strain data to minimize potential error generally associated with the fitting procedure. Poor fit may indicate either incorrect models were chosen or data insufficiency [18]. It should be noted that the strain energy potential is defined as the strain energy stored per unit of reference volume expressed as a function of strain at that point in the material. The mathematical form of the polynomial strain energy potential, *U* [29] used in this study is shown in Equation (1):

(1) $$U=\;\overset{N}{\underset{i+j=1}{\sum }}C_{ij}{\left({\bar{I}}_{1}-3\right)}^{i}{\left({\bar{I}}_{2}-3\right)}^{j}+\;\overset{N}{\underset{i=1}{\sum }}\frac{1}{D_{i}}{\left(J_{el}-1\right)}^{2i}$$

where *N* represents the polynomial order with a maximum value of 6, ${\bar{I}}_{1}$ and ${\bar{I}}_{2}$ signify the strain invariants, $C_{ij}$ is the material parameter controlling shear behaviour and $D_{i}$ is the material parameter to induce compressibility. First portion in the equation represents the deviatoric term, simulating shape distortion expected under applied stress and the second portion represents the volumetric term simulating volume change under applied stress. The final form of the above equation will vary and encompass different constants depending on the choice of the SEF. Table 1 tabulates the various SEFs available in ABAQUS. It should also be noted that the Mooney Rivlin, Ogden and Yeoh functions are typically classed as the phenomenological models, where the stress–strain response is characterized without giving consideration to the microstructure of rubber, while the other models are physically motivated models [29]. In this study, all the models listed in Table 1 are used to analyse their efficiency and suitability to predict the stress–strain response of ROPS. Note that Van der Walls and Ogden functions are not stable for the strain range studied in this work and, hence, are not considered for further analysis.

It should be noted that the two-dimensional and axisymmetric models were not adopted in this study owing to the limitations associated with these methods [34]. Moreover, three-dimensional models are proved to be more efficient [28,35]. The 3D finite element model developed for ROPS composite in this study involves 3 key components—(a) top loading platen, (b) bottom loading platen and (c) the deformable ROPS composite in between. The platens were modelled as rigid parts, to save on computational time. Tie constraints were established at the interface of the ROPS composites and rigid surface to simulate the actual experimental conditions and prevent slipping of the ROPS specimen while loading. After defining the required parts, utmost importance was then given to the hyperelastic material definition of the ROPS composites in this study. To start with, ROPS test pieces alone were subjected to modal frequency analysis to evaluate their lowest natural frequency, which is critically important in assessing the stability of the ROPS specimen in the model. The value was found to be 5.076 Hz and the loading frequency is apparently less than 1/5th of this lowest natural frequency and, hence, a static general step with nonlinear geometry ON, was deemed to be used in simulating the compressive loading [36]. The bottom rigid loading platen was assigned with a fixed boundary condition. The examples of a representative model used in this study highlighting the loading platens and ROPS composite is shown in the Figure 3e. Loading and unloading cycles were simulated using a displacement time history corresponding to strain rate around 50%. Meshing was conducted using hybrid elements of type C3D8RH with enhanced hourglass control in order to account for excessive deformation. In most of the hyperelastic analysis for rubber and polymers, hybrid elements of type C3D8RH were found to be used successfully [27,32,35]. The mesh density of hexagon- and octagon-shaped ROPSs was made relatively high near the edge and sharp corners, to reduce the presence of distorted elements. This was also selected to improve accuracy of the simulation results. Mesh verification indicated no analysis error and the number of elements generated were 1367 for the circular samples and ranged from 2600 to 7500 for the polygon-shaped samples. The size of a single mesh unit was in the range of 1 to 2.1, as observed in the mesh optimization studies.

## 4. Results and Discussion

**Figure 2:**
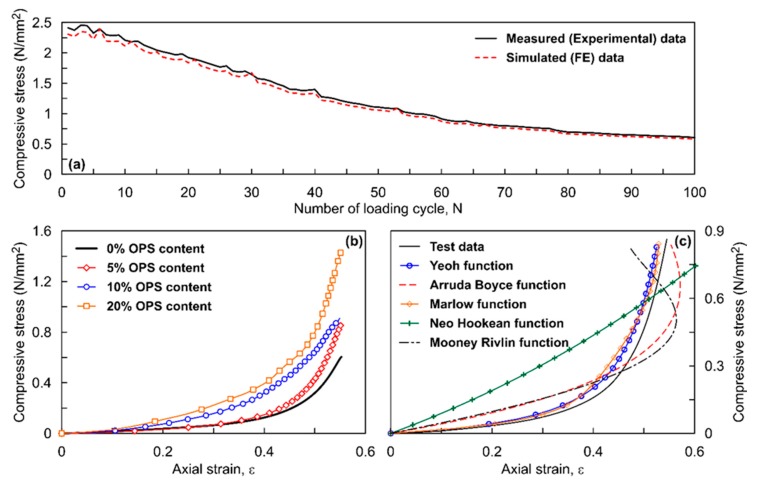
Typical response of circular shaped ROPS specimen to compressive loading: (**a**) variation of compressive stress with N; (**b**) effect of increasing OPS content on measured stress–strain response of ROPS samples in loading stage; (**c**) curve fitting of different SEFs with the experimental data.

Figure 2a shows the typical response expressed as stress versus number of loading cycles (N), both measured in the laboratory experiments and the FE simulations obtained for the circular shaped ROPS specimen. The figure shows the data over the first 100 cycles. As evident in the figure, there is a decrease in the compression stresses (σ_comp_) resisted by the ROPS specimens, up to around 70 cycles in this example, whereas σ_comp_ values stabilized with minimal decrease or variation in the stress values with increasing N. Thus, the data from the 70th cycle has been used for comparison and analysis in this study. Overall, a good agreement was observed within the experimental and FE simulation data, with minor error (discussed later in this section).

### 4.1. Evaluation of the Material Coefficients

Figure 2b shows a typical stress–strain response for the circular ROPS specimen in the loading cycle (only first half of the loading-unloading loop is shown). Observations from this figure show that the compressive stresses range between 600 kN/m^2^ for pure rubber (0% OPS) and 1440 kN/m^2^ for ROPS with 20% OPS content. This clearly explains the contribution of OPS content on the strength improvement in the ROPS composites. The corresponding Young’s modulus as well ranges between 5000 kN/m^2^ to 8000 kN/m^2^, while the Poisson’s ratio value of 0.45 was obtained analysing the experimental data. Nonetheless, the main purpose of the experimental stress–strain data in this study was to provide a numerical approximation in order to derive the stable and suitable SEFs for hyperelastic modelling of the ROPS specimens. Thus, the uniaxial test data measured for every ROPS specimen experimented, were fed as input into the ABAQUS, following which different available SEFs derived were evaluated based on their best fit with the experimental curves. Figure 2c shows a representative example of curve fit of different SEFs with the experimental data obtained for circular ROPS with 5% OPS content. Note that curve fits with similar trends were obtained for other ROPS types as well. As listed in the figure, five relevant SEFs available in the ABAQUS were tested against the experimental data. It can be observed that the Yeoh function and Marlow function show a relatively good comparison with the experimental data. The Arruda Boyce function and Mooney Rivlin function show moderate comparison (at ε < 20% only), while the Neo Hookean function is clearly not in match. However, these five functions were deemed stable and used to estimate the material coefficients (see Table 1 for details of the functions and coefficients/constants) for each function. Table 2 tabulates the material coefficients obtained for the five stable SEFs for the circular ROPS specimens in this study. Similar procedure was followed in estimating the material coefficients for square, hexagon- and octagon-shaped ROPS specimens as well. The hyperelastic ROPS models were then established based on these material coefficients, used in pertinent equations of the hyperelastic material model.

### 4.2. ROPS Model Simulations

**Figure 3:**
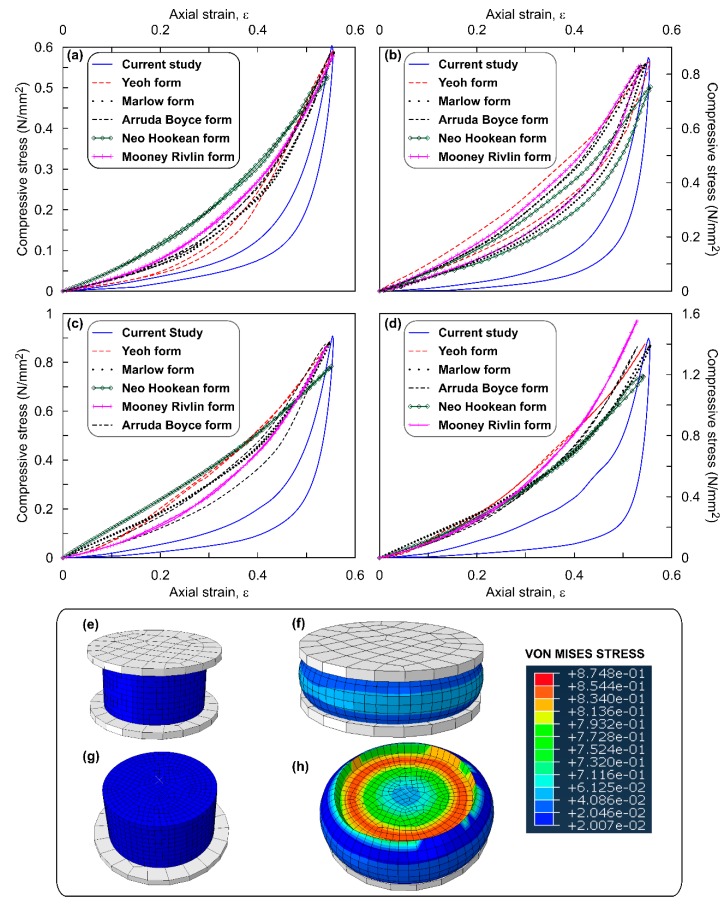
Stress–strain response of circular ROPS composites: (**a**) 0% OPS; (**b**) 5% OPS; (**c**) 10% OPS; (**d**) 20% OPS content; (**e**) undeformed ROPS model; (**f**) deformed ROPS model; (**g**) top-view of undeformed model at ε ≈ 50%; (**h**) top-view of deformed model with stress contours at ε ≈ 50%.

Figure 3a–d show the stress–strain response of the circular ROPS specimens simulated using the hyperelastic functions for all four OPS contents. Figure also highlights the comparison of these simulation outputs with the experimental output. Observations from the figure clearly evince that all the SEFs match with the experimental curve—exhibiting similar concave upward pattern. However, for 10% and 20% OPS content alone, the Neo Hookean function represents a linear pattern. Similar patterns are evident irrespective of the varying OPS contents. The maximum compressive stress (σ_max_), using the Yeoh function ranged between 590 and 1400 kN/m^2^ which is in good comparison with the experimental data (604–1438 kN/m^2^). Likewise, the range of σ_max_ using other models are, 585–1370 kN/m^2^ for Arruda Boyce, 580–1370 kN/m^2^ for Marlow, 579–1538 kN/m^2^ for Moone, and 525–1185 kN/m^2^ for Neo Hookean functions. Overall, an increase in σ_max_ values was witnessed with increasing OPS contents, which also agrees with the observations from the experiments. For example, σ_max_ value for the ROPS specimens with 20% OPS was approximately 2.4 times higher than that of ROPS specimens without OPS content. Increasing σ_max_ values can be correlated to indicate higher absorption of the impact energy [28]. This conceptually attributes to the distinct property of OPS which imparts increasing stiffness and hence improved strength and shock absorption. Table 3 tabulates the percentage error observed in the σ_max_ values estimated from the model simulation with that of the experiments. A lower error was observed in the case of the Yeoh model, thus justifying its suitability in simulating the stress–strain response of the ROPS and similar composites. Next to Yeoh, Marlow and Arruda Boyce functions were found preferable with an error less than 5%. The Mooney Rivlin function though exhibited error less than 7%, an overestimation of stress value was observed in ROPS composites with 20% OPS content, thus making this function only moderately suitable. The error with Neo Hookean model was relatively higher, thereby proving to be inappropriate for modelling ROPS composites to this end.

The comparison of percentage error values across different OPS contents reveals that ROPS composites with 20% OPS have higher error magnitudes in comparison to 0%, 5% and 10% OPS contents. This can also be related to the random orientation of OPS considered in this study. Thus, further studies are recommended to check for anisotropy at higher OPS contents and orientations. Figure 3g,h show the contour plots of the Von Mises stress observed for a circular ROPS specimen when strain (ε) is approaching 50%. Figure 3h reveals higher stress transfer directly below the loading ram, at the interface of the loading ram and ROPS’s surface, migrating gradually towards the internal zones. Further, deformations ranged from 0 for un-deformed material to 11.01 mm for deformed specimen. ROPS specimen did not show a specific failure type and maximum permanent deformation after 100 loading cycles was observed at 4%, which corresponds to 0.70 mm.

**Figure 4:**
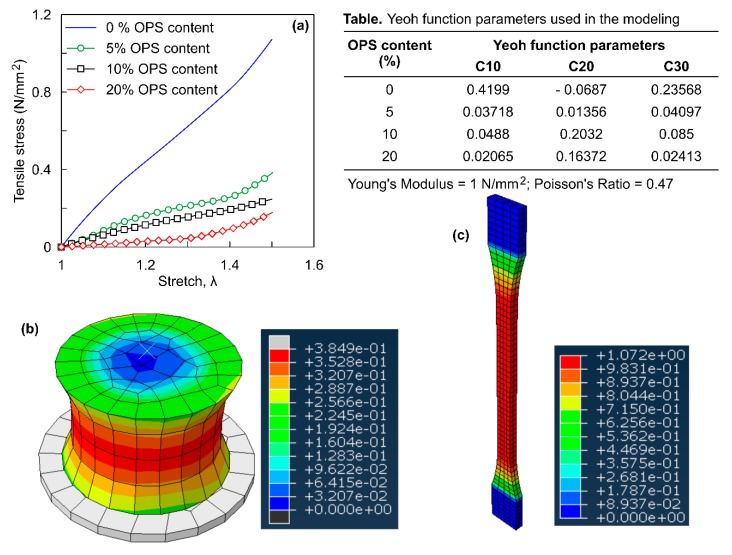
FE simulation of specimen under tension: (**a**) stress-stretch response of ROPS specimen; (**b**) tensile stress contours of ROPS with 5% OPS; (**c**) tensile stress contour of dumbbell shaped rubber.

The main purpose of the ROPS composites considered in this study is to resist the energy generated due to impact or compressive type loads. Nonetheless, for validations purposes tensile testing of the ROPS composites were also carried out. The uniaxial stress stretch (tensile) behaviour of the ROPS composites were simulated using hyperelastic (aided with the Yeoh function) modelling which is evident as most suitable function for ROPS circular specimens. A dumbbell-shaped specimen and a circular specimen of ROPS was considered for this purpose. The geometry of the dumbbell shaped specimen used in the tensile test FE simulation for pure rubber was 150 (*l*) × 25 (*w*) × 3 (*t*) mm, similar to procedure presented in Sahari and Maleque [15]. The material parameters used for the simulation are listed in the table highlighted in Figure 4. The loading procedure basically involved stretching the specimens to 1.5 times the original specimen length. Meshing involved the hybrid elements, followed by convergence analysis to get the most appropriate output. Figure 4a shows the tensile stress for ethylene propylene diene monomer (EPDM) based rubber without OPS is 1.072 MPa. This value evidently is in the range of the tensile stresses of unfilled EPDM as presented in Milani and Milani [37], thus validating the modelling procedure and reliability of the simulation results. Figure 4c shows the tensile stress contours obtained for dumbbell shaped specimen with no OPS content. It should be noted from the work presented by Milani and Milani [37] that the Mooney Rivlin simulations of the commercial rubbers with carbon black fillers showed stiff curves with quick increase in uniaxial tensile stress as compared to that of the unfilled rubber. However, simulations in this study shows an opposite behaviour with the pure rubber (with no filler/OPS) achieving higher strength more quickly as compared to its OPS counterparts.

The values of tensile stresses obtained for the circular ROPS composites are less than their compressive stress values, in spite of maintaining similar specimen dimensions. Quantitatively, the tensile stresses were approximately less than 0.5 times the compressive stresses for the same specimen. This is because, the effective area that resists the tensile force gets reduced with increasing OPS content. This can be correlated to perforations induced in steel plate, which are likely to reduce the force transmitted [38]. The presence of OPS in rubber matrix reduces the tensile force transmitted in similar manner, since they can disperse only compressive loads and cannot transfer any tensile force. Hence, the maximum force at yield also gets reduced, leading to lower values of tensile stress. Moreover, studies employing fibre-rubber reinforcements also demonstrates similar reductions in tensile strength with increase in the fibre content [39,40,41].

The suitability of the hyperelastic models used for circular ROPS specimens was further checked for possible stability limitations that might arise due to varying shape (surface) of the composites. It is for this reason that three additional shaped ROPS specimens (square, hexagon and octagon) were cast and tested in the laboratory. It should be noted that the similar procedure and methodology was adopted in the simulations and experiments conducted on all other shaped ROPS specimens as well.

**Figure 5:**
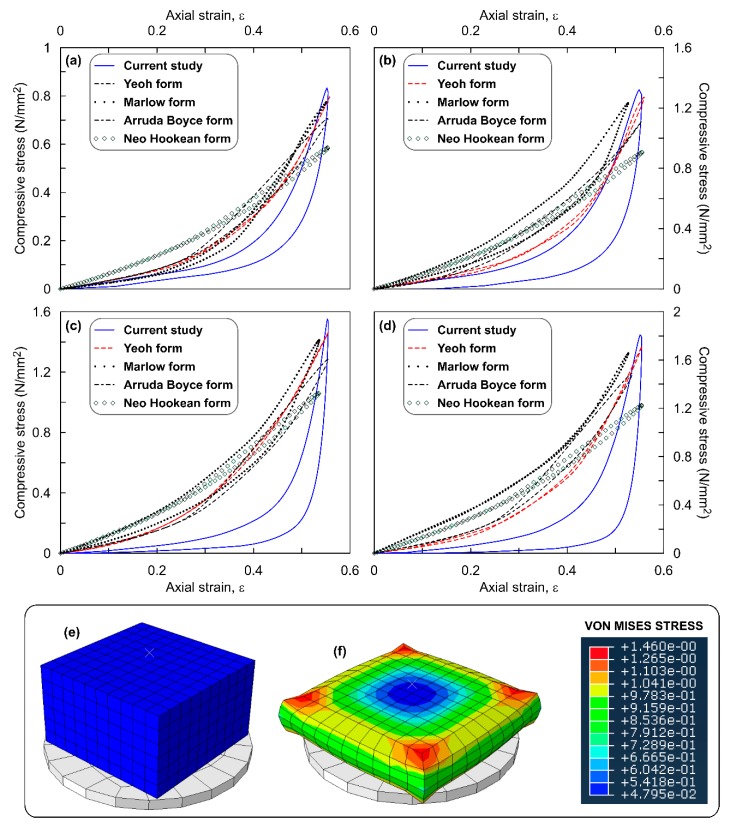
Stress–strain response of square shaped ROPS specimens: (**a**) 0% OPS; (**b**) 5% OPS; (**c**) 10% OPS; (**d**) 20% OPS; (**e**) un-deformed model; (**f**) deformed model with stress contours at ε ≈ 50%.

Figure 5a–d shows the comparison of stress–strain response simulated with that of the data measured in the experiments on square ROPS specimens with OPS content of 0%, 5%, 10% and 20% respectively. Observations from the figure highlight similar patterns of the simulation outputs in comparison with the experiments. Yeoh model was found to best illustrate the experimental data with only 5–6% error. Marlow and Arruda Boyce models occupy the next level with error ranging up to 9.6% and 17.80%, respectively. However, these error values are on the higher side as compared to that observed with the circular ROPS specimens (7%). This increase in error can be attributed to element distortions at the sharp edges, following which these elements upon experiencing excessive distortion may reach a scenario which cannot be modelled using hyperelasticity function. Mooney Rivlin function which was found to be stable for circular shaped ROPS was found unstable in the case of square shaped ROPS specimen. The material convergence of Mooney Rivlin is believed to have been influenced by the varying stress–strain conditions induced due to the sharp edges. This is also proved by the contour plots in Figure 5f, where the maximum stress is concentrated at the sharp corners and not at the centre unlike in circular shaped ROPS specimens. The stress distribution along the edges were higher than that of the elements directly below loading ram. Other than this, the trends of poor predicting accuracy of Neo Hookean, increasing maximum stress values with increasing OPS content, and having higher error values for 20% OPS content ROPS specimens remained unchanged for the square shaped ROPS as well. Moreover, the maximum deformation recorded was 10.67 mm and the permanent deformation imparted is 4.44% (0.89 mm).

**Figure 6:**
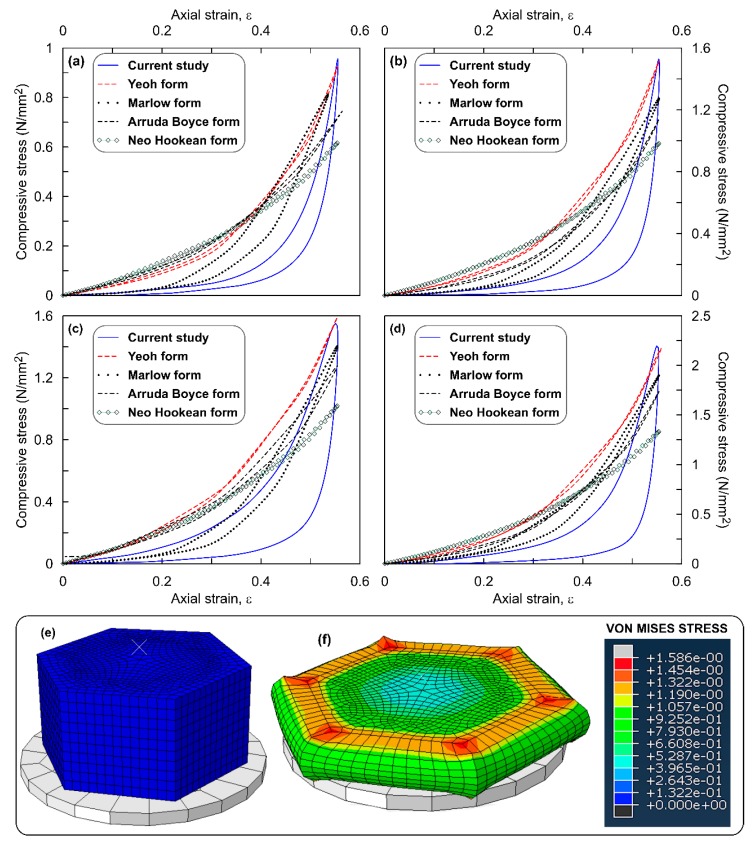
Stress–strain response of hexagon shaped ROPS specimens: (**a**) 0% OPS; (**b**) 5% OPS; (**c**) 10% OPS; (**d**) 20% OPS; (**e**) un-deformed model; (**f**) deformed model with stress contours at ε ≈ 50%.

Figure 6a–d shows the comparison of stress–strain response simulated with that measured in the experiments on hexagon shaped ROPS specimens with OPS content of 0%, 5%, 10% and 20%, respectively. The best estimation was observed to have been achieved considering the Yeoh function with a 3–6% error, followed by Marlow and Arruda Boyce functions with 15% and 22% error, respectively. These error values are higher than square shaped ROPS specimens, as the number of edges/corners is high in comparison to square specimens. Contour plots in Figure 6f support this statement, which shows the maximum stress concentrations occurring at the sharp corners of the loading surface and along edges. The permanent deformation recorded was 3.8% (0.76 mm).

**Figure 7:**
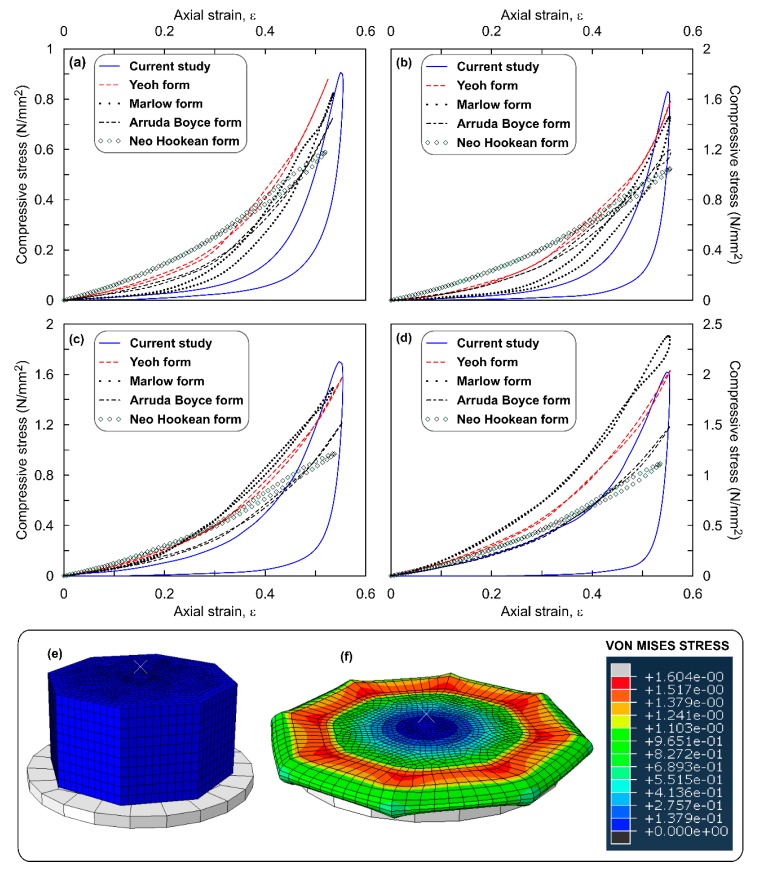
Cyclic stress strain response of Octagonal samples: (**a**) 0% OPS; (**b**) 5% OPS; (**c**) 10% OPS; (**d**) 20% OPS; (**e**) un-deformed model; (**f**) deformed model with stress contours at ε ≈ 50%.

The behaviour of octagonal samples was very similar to that of observed in square and hexagon shaped ROPS specimen, except that the Marlow function was found to overestimate the σ_max_ values in ROPS with 20% OPS content. Figure 7a–d shows comparison of simulated stress–strain response with that of the experimental data. Yeoh was the most appropriate with a 3–7% error, followed by the Marlow and Arruda Boyce functions with 16.7% and 20% error, respectively. The contour plots in Figure 7f show the occurrence of σ_max_ at sharp corners which migrate further to the edges, similar to square- and hexagon-shaped specimens. Permanent deformation recorded was 4.3% (0.86 mm).

### 4.3. Effect of Shape on the Stress–Strain Response of ROPS Specimens

Overall observations from Figure 3, Figure 4, Figure 5, Figure 6 and Figure 7 leads to an understanding that the increasing number of sharp corners and edges results in an increased error in the σ_max_ values predicted using the hyperelastic SEFs used in this study. This may be due to the element distortions, which is perhaps unavoidable with ROPS shapes containing edges and corners, though the mesh verification process revealed zero error. Nevertheless, the hyperelastic model aided by the Yeoh function was able to predict well the stress–strain response of the ROPS specimens for all shapes. This statement is justified based on the lower error in σ_max_ values (−7% to 0.7%) between predicted and experimental data. Mesh optimization considering different trials may be used to further reduce the error where required.

Another key factor, which is of major interest in this study, is the variations in stress distribution across the range of ROPS shapes considered. Especially, the corners and edges associated with the polygon (square, hexagon and octagon) shaped ROPS specimens revealed to have a major effect on the stress distribution pattern when compared to that in the circular shaped ROPS specimens. Literature also highlight the presence of corners and/or edges to increase the stress concentration in hexagon and octagon specimens, which are mostly located close to the corners as compared to that of circular and/or square shaped specimens [42]. Having said this, there is yet one similarity observed in the stress distribution—higher stress concentrations are predominantly noted at close proximity to the loading surface than that at the lower portion or lateral surface of ROPS specimens. This can be explained with the transfer of stresses to the adjacent areas to have not yet initiated, particularly due to insufficient time owing to the loading frequency (0.8 Hz in this study). Nonetheless, higher stress concentrations at the circumference and edges may relate to the lateral movement (pushing effect) of OPS samples dispersed in the rubber matrix during maximum loading. Since the load transfer mechanism in the OPS (dome-shaped structures) is mainly by contact between the adjacent OPS, thereby leading to possible pushing effect, thus resulting in higher stresses at the specimen periphery.

The next important factor considered in this analysis was the variation in stress intensities. Though four different shapes were used in casting ROPS specimens, an equal loading area was devised to ensure similar loading transfer perhaps. This was considered essential to ensure equivalent stress intensities. However, the stress intensities obtained or observed were not similar and were found to vary with varying shapes of the ROPS. Octagon- and hexagon-shaped specimens did exhibit highest stress intensities, followed by square and circle shaped ROPS. This difference can be related to the effect of shape factor on the vertical stiffness of ROPS composites. The shape factor is typically defined as the ratio of loaded top area to the lateral surface area available for bulging. The shape factor plays an important role in determining the compression behaviour and the associated stress intensities [43]. The effect of shape factors on the vertical stiffness of ROPS specimens should also be evaluated in order to understand the optimum range of shape factor that can help acquire the required range of vertical stiffness for the end application of the ROPS specimens.

**Figure 8:**
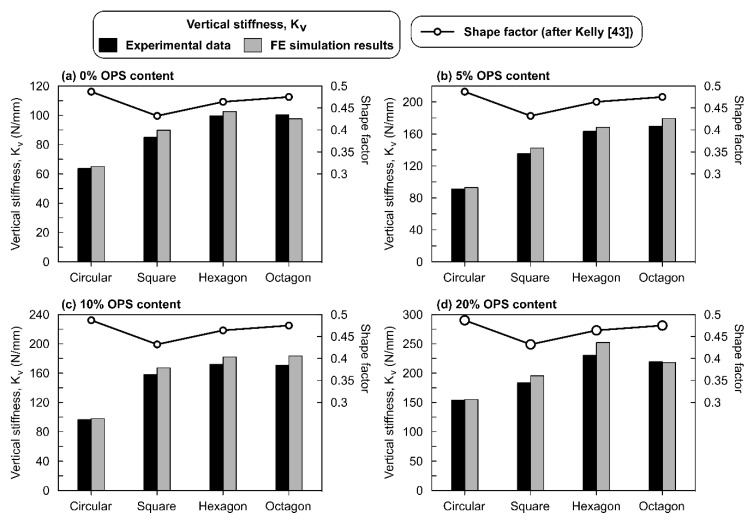
Variation of the shape factor values and vertical stiffness obtained for different shapes in this study: (**a**) 0% OPS; (**b**) 5% OPS; (**c**) 10% OPS; (**d**) 20% OPS.

Figure 8a–d shows the variation of shape factor and the associated vertical stiffness of all of the four shapes used for the ROPS specimens in this study. Observations from the figures show that circular specimens with a shape factor of 0.48 have the lowest vertical stiffness value. While the increase in OPS content from 0% to 20% increases the vertical stiffness values of circular specimen. Nonetheless, this increase is consistently low in comparison with its counterpart polygon-shaped ROPS specimen. Square-shaped ROPS with a shape factor of 0.42 recorded stiffness values higher than those of circular samples and there was a continuous increase in the vertical stiffness value with the increase in OPS content. Likewise, hexagon-shaped specimens with shape factor of 0.46 recorded the highest vertical stiffness. The octagon-shaped specimen (shape factor of 0.475) showed vertical stiffness values similar to hexagon shaped specimen up to 10% OPS content. However, in the 20% OPS condition, a reduction in the octagon’s vertical stiffness was observed.

Hypothetically, within an optimum range of shape factors, the vertical stiffness is expected to increase with increasing shape factor values [43]. This trend can be observed in the case of square- and hexagon-shaped specimens, while minor variation was observed in the octagon-shaped specimen. However, the circularly-shaped specimen, having the highest shape factor did not show such a trend, indicated by their lower vertical stiffness values. These observations perhaps indicate that the shape factor in the range of 0.42–0.47 can be considered as optimum for ROPS. Moreover, a typical elastomer with a higher shape factor is expected to behave differently as compared to that of the lower shape factor samples [44]. Based on the trends observed in this study, it is apparent for the ROPS specimen with a shape factor greater than or equal to 0.48 can be classed as higher shape factor composites (SH) and all the polygonal-shaped specimens with shape factor values less than 0.47 can be classed as lower shape factor samples (SL). Another key observation which supports this classification is the high vertical stiffness occurring in SL samples as compared to that of SH samples, which is found compatible with earlier studies [42,44]. SH samples are believed to have higher horizontal stiffness, which is yet to be studied in the case of the ROPS composites. The minor deviations observed in the octagon shaped specimen are due to its shape factor value approaching the SH type. From these stiffness and shape factors studied, the vertical stiffness values of polygonal shaped specimens are comparable to that of the carbon fibre-reinforced rubber samples [44].

## 5. Conclusions

This paper presents a pioneering investigation using the finite element hyperelastic model and its suitability in predicting the stress–strain response of ROPS composites with different OPS contents and shapes. The compressive strain controlled tests to ε ≈ 50% at 0.8 Hz frequency were conducted in the laboratory to evaluate the required material coefficients, further used as input to the FE simulations. The measured σ_max_ values ranged 860–2340 kN/m^2^, while the Young’s modulus ranged between 5000 kN/m^2^ and 20,000 kN/m^2^. As anticipated, the OPS-rubber matrix was observed to partially consume (dampening effect) the stress at every loading cycle as evident from the loading-unloading loop. The experimental stress–strain data was used to simulate the material coefficients of selected SEFs which were further utilized to simulate the behaviour of ROPS specimen using hyperelastic models. The Yeoh model (error less than 2.7% for circular shaped ROPS) was found to perform best in comparison with the experimental results and, thus, the most stable and suitable hyperelastic model to this end. The Marlow (error less than 4.66% for circular shaped ROPS) and Arruda Boyce (error less than 4.74% for circular shaped ROPS) models were amongst the next alternatives to perform better. The error in predicting the response was found to increase for other shaped ROPS specimen irrespective of the OPS contents considered in this study. Nevertheless, the highest error using the Yeoh model was observed around 7%, which is deemed reasonable. On the other hand, simulations using tensile loads highlight that the presence of OPS in rubber matrix most likely reduces the tensile force transmitted, while they effectively can disperse compressive loads. Not limiting to simulating the stress–strain response alone, the five SEFs selected were tested to also ascertain the effect of varying composite shapes. The shape effect was further compared with the literature outcomes. Particular emphasis was laid to compare and assess the response simulated in this study with that observed in the literature on similar composites. The specific responses and trends showed good comparison with the literature and experimental measures, thereby adding further confidence in the simulation procedure used in this paper. Thus, we conclude that adopting the Yeoh model or a similar material law would be an ideal approach to estimate the stress–strain response of the ROPS or equivalent rubber composites. Having said this, and owing to the scarce literature or knowledge till date, we recommend further research with focus on the ROPS or equivalent composites—both experimental and numerical simulation studies involving more than one deformation mode. Nonetheless, we strongly believe that this paper contributes in understanding the immediate need for selecting the most appropriate function for hyperelastic models to simulate the behaviour of ROPS for effective vibration control applications.

## Figures and Tables

**Table 1:** Summary of the SEFs considered in this study [29].

The Mooney Rivlin function is obtained by setting N = 1 in Equation (1). This function is moderately suitable for large strains in uniaxial tension and shear distortion [20,30]. The mathematical form of this function is $U=C_{10}\left({\bar{I}}_{1}-3\right)+C_{01}\left({\bar{I}}_{2}-3\right)+\frac{1}{D_{1}}{\left(J_{el}-1\right)}^{2}$ Unlike other polynomial functions, the Mooney Rivlin function is predominantly dependent on the first strain invariant $({\bar{I}}_{1}$), thereby predicting complex material behaviour even with limited test data and strains less than 100% [31].
The Neo Hookean function is a first order reduced polynomial form obtained by equating $C_{01}$ to zero in Mooney Rivlin function. The function has the form $U=C_{10}\left({\bar{I}}_{1}-3\right)+\frac{1}{D_{1}}{\left(J_{el}-1\right)}^{2}$ Neo-Hookean functions are mostly suitable for small strain applications [32].
The Yeoh function is a third order reduced polynomial function by Yeoh [22]. Unlike above 2 functions, Yeoh model is suitable for wider range of strains and simulate different deformation modes even with limited test data. This function is expressed as $U=\;\overset{3}{\underset{i=1}{\sum }}C_{i0}{\left({\bar{I}}_{1}-3\right)}^{i}+\;\overset{3}{\underset{i=1}{\sum }}\frac{1}{D_{i}}{\left(J_{el}-1\right)}^{2i}$
The Arruda Boyce function [33] is expressed as $U=\;\mu \sum_{i=1}^{5}\frac{C_{i}}{\lambda_{m}^{2i-2}}C_{ij}\left({\bar{I}}_{1}{}^{i}-3^{i}\right)+\frac{1}{D}\left(\frac{J_{el}{}^{2}-1}{2}-\;ln\;J_{el}\right)$ is a hyperelastic material law. Where, $C_{1}=\;\frac{1}{2}\;,{\;C}_{2}=\;\frac{1}{20}\;,{\;C}_{3}=\;\frac{11}{1050}\;,{\;C}_{4}=\;\frac{19}{7000}{\;\mathrm{and}\;C}_{5}=\;\frac{519}{673750}$. Arruda Boyce depend on first invariant (${\bar{I}}_{1})$; suitable for wide strain range and limited material data
The Marlow form is expressed as $U=U_{\mathrm{dev}}\left({\bar{I}}_{1}\right)+U_{\mathrm{vol}}(J_{\mathrm{el}})$, where ${\bar{I}}_{1}$ is the first strain invariant, $J_{el}$ is the elastic volume ratio and $U_{dev}\;and$ $U_{vol}$ represent deviatoric and volumetric part of strain energy, respectively. The Marlow form simulates reasonable behaviour even in cases having single test data.

**Table 2:** Material coefficients of stable strain energy functions of circular ROPS specimens.

OPS (%)	Mooney Rivlin		Neo Hookean	Yeoh			Arruda Boyce	
	C10	C01	C10	C10	C20	C30	µ	λ_m_
0	−0.0255	0.0412	0.0419	0.0295	−0.0042	0.0088	0.0498	2.2196
5	0.0270	0.0006	0.0807	0.0293	−0.0030	1.3414	0.0548	3.0235
10	0.0293	0.0672	0.1281	0.0705	0.0406	−0.0073	0.1226	1.0911
20	0.0513	0.1439	0.1516	0.0894	0.1350	−0.0457	0.2397	1.1606

**Table 3:** Summary of σ_max_ (in kN/m^2^) and error for circular ROPS specimens.

OPS Content	SEF Type	σ_max_—Simulation	σ_max_—Experimental	Error (%)
0%	Yeoh	581.35	604	−2
	Marlow	591.92		−3.8
	Arruda Boyce	585.88		−3
	Mooney Rivlin	579.84		−4
	Neo Hooke	525.48		−13
5%	Yeoh	828.72	862	−2.2
	Marlow	843.173		−3.9
	Arruda Boyce	837.174		−2.9
	Mooney Rivlin	824.41		−4.4
	Neo Hooke	745.199		−13.6
10%	Yeoh	867.82	909	−2.5
	Marlow	885.911		−4.5
	Arruda Boyce	874.0035		−3.9
	Mooney Rivlin	852.18		−6.3
	Neo Hooke	775.9224		−14.6
20%	Yeoh	1370.989	1438	−2.7
	Marlow	1399.6054		−4.7
	Arruda Boyce	1369.83		−4.7
	Mooney Rivlin	1538.66		7
	Neo Hooke	1185.631		−17.6
